# The Influence of Maternal Diet in Late Pregnancy on Malondialdehyde and Cortisol Levels in Maternal and Cord Blood

**DOI:** 10.3390/nu17061077

**Published:** 2025-03-19

**Authors:** Mai Quynh Nguyen, Kinuyo Munakata, Midori Natsume, Yoshitaka Nakamura, Hiroshi Miyabayashi, Nobuhiko Nagano, Ichiro Morioka

**Affiliations:** 1 Wellness Science Labs, Meiji Holdings Co., Ltd., Tokyo 192-0919, Japan; quynhmai.nguyen@meiji.com (M.Q.N.); midori.natsume@meiji.com (M.N.); 2 R&D Division, Meiji Co., Ltd., Tokyo 192-0919, Japan; 3 Department of Pediatrics, Kasukabe Medical Center, Saitama 344-8588, Japan; miyabayashi@dr.memail.jp; 4 Department of Pediatrics and Child Health, Nihon University School of Medicine, Tokyo 173-8610, Japan

**Keywords:** pregnancy, maternal diet, oxidative stress, maternal health, child development

## Abstract

**Background/Objectives:** Research suggests that diet influences oxidative stress status in pregnant women and is related to their stress and depressive symptoms. This study aimed to investigate how maternal diet during late pregnancy affects oxidative stress status, maternal stress, depression, and fetal physical development. **Methods:** This study included 58 mother–child pairs. Dietary intake, depressive symptoms, and clinical information were obtained through questionnaires and clinical records. Maternal and cord blood concentrations of malondialdehyde (MDA), paraoxonase-1, platelet activating factor-acetylhydrolase, and cortisol were measured using spectrophotometric and enzyme-linked immunosorbent assays. **Results:** Maternal serum MDA levels were inversely associated with nut consumption (β = −0.40, *p* = 0.01) and positively associated with vitamin B2 (β = 2.43, *p* = 0.04) and manganese intake (β = 0.44, *p* = 0.02). Fruit consumption was positively associated with Center for Epidemiologic Studies Depression Scale score (β = 0.35, *p* = 0.03). Intakes of beans (β = −3.37, *p* = 0.04), vitamin B1 (β = −738.92, *p* = 0.04), vitamin B6 (β = −562.21, *p* = 0.04), vitamin C (β = −4.75, *p* = 0.009), iron (β = −106.63, *p* = 0.03), and copper (β = −863.31, *p* = 0.01) were inversely associated with maternal serum cortisol level, whereas dairy intake (β = 1.45, *p* = 0.003) showed a positive association. Cord plasma cortisol levels were inversely associated with the consumption of other vegetables (β = −2.89, *p* = 0.02). **Conclusions:** The findings encourage further research towards the refinement of dietary guidelines for pregnant women and recommendations for expecting mothers.

## 1. Introduction

Oxidative stress occurs when there is an imbalance between the production and accumulation of free radicals, such as reactive oxygen species (ROS), and their detoxification by biological systems in cells and tissues [1]. ROS, including superoxide radicals (O_2_•−), hydrogen peroxide (H_2_O_2_), hydroxyl radicals (•OH), and singlet oxygen (1O_2_), can have harmful effects on important cellular structures like proteins, lipids, and nucleic acids when their production increases [1]. Oxidative stress is associated with several diseases, including cancer, diabetes, metabolic disorders, atherosclerosis, and cardiovascular diseases [1], and its role in depression is well known [2]. During pregnancy, oxidative stress can lead to complications such as gestational hypertension, nausea and vomiting, gestational diabetes, and pre-eclampsia [3]. Results from a cohort study suggest that high levels of oxidative stress are associated with postpartum depression [4]. Some recent publications emphasize the relationship between inflammation, which can trigger the production of ROS leading to oxidative stress, and perinatal depression [5,6]. Oxidative stress during pregnancy is also proposed to affect fetal development [3] and child development, correlating with birthweight [7] and emotional and behavioral problems in early childhood [8].

Research into the mechanisms underlying oxidative stress has received considerable attention. Studies on the relationship between dietary patterns, specific food types (e.g., vegetables/fruits), and/or single nutrients (e.g., fats and vitamins) with oxidative stress markers suggest that diet affects oxidative stress status in pregnant women [9,10,11,12]. It has also been reported that diet during pregnancy is closely related to maternal stress and depressive symptoms, with an unhealthy diet being associated with higher levels of depressive symptoms and stress [13,14]. Further, there is growing attention on the importance of diet during different phases of pregnancy, with some reports emphasizing the significance of diet in late pregnancy—the third trimester [15,16].

In the present study, we aimed to investigate how maternal diet during late pregnancy affects oxidative stress status, maternal stress and depression, and fetal physical development. We focused on late pregnancy consumption of a wide range of food types and nutrients that are reported to influence pregnancy and fetal development: vitamins [17] and minerals [18]. For oxidative stress status evaluation, we measured blood levels of MDA, a well-recognized oxidative stress marker [9]. We also evaluated blood levels of antioxidants, including paraoxonase-1 (PON-1) [19] and platelet-activating factor-acetylhydrolase (PAF-AH) [20], and blood cortisol levels. Maternal depression during late pregnancy was assessed using the Japanese version of the Center for Epidemiologic Studies Depression Scale (CES-D) [21]. Fetal physical development was evaluated based on standard deviation scores of birth weight, birth height, and birth head circumference. We hypothesize that the late-pregnancy diet is associated with oxidative stress status, which, in turn, is linked to both maternal and fetal health. By managing dietary intake, it is possible to control oxidative stress status and assure healthy pregnancy. With our findings, we hope to contribute to the encouragement of further research to clarify the relevance and aim towards refinement of dietary guidelines for pregnant women and to give direction for recommendations for expecting mothers.

## 2. Materials and Methods

### 2.1. Study Design and Subjects

In this study, women aged 18 years or older who visited and received medical treatment at the Department of Obstetrics and Gynecology at Kasukabe Medical Center were recruited from October 2022 to February 2024. Eligible subjects were women between 28 and 40 weeks pregnant at the time of obtaining informed consent, along with their children. Women judged by the principal investigator to be inappropriate as study subjects and those with multiple pregnancies were excluded. In the case of multiple pregnancies, there are blood and nutrition survey data for one mother, but there are multiple cord blood and child data, so the same mother’s data must be used multiple times in evaluation. To avoid that, multiple pregnancies were excluded from the target at the planning stage. The study population included 58 women and their newborns.

After obtaining informed consent from the study participants, the following information was collected: (1) at enrollment: age, pre-pregnancy body mass index (BMI), weight before pregnancy, complications (such as presence or absence of pregnancy-induced hypertension or gestational diabetes), smoking and alcohol intake during pregnancy, maternal dietary habits, and depressive symptoms during late pregnancy; (2) at delivery: mode of delivery, weight at birth, placental weight, and blood samples. For newborns, data collected at delivery included gestational age, birth weight (BW), birth height (BH), head circumference, Apgar score, and cord blood samples.

### 2.2. Blood Analyses

Maternal and umbilical cord blood samples were collected. For maternal blood sampling, 5 mL of venous blood was collected in the morning on an empty stomach. For the infant’s blood, at least 5 mL of umbilical artery blood was collected at birth from the delivered placenta and umbilical cord. Samples were handled with care to minimize the breakdown. Serum separation was performed using a centrifuge with centrifugation at 3500 rpm for 5 min. Samples were stored at −80 °C until analysis.

Commercial kits were used to measure blood cortisol (#50191, IBL International GmbH, Tecan Group, Hamburg, Germany), PON-1 (ARG81509, Arigo Biolaboratories, Taiwan, ROC), and PAF-AH (ELH-LPPLA2, RayBiotech, Peachtree Corners, GA, USA) levels. Blood MDA concentration was measured using a commercial kit (NWK-MDA01, Northwest Life Science Specialities LLC, Vancouver, WA, USA), and data were analyzed using the manufacturer’s online platform (https://www.nwlifescience.com/sg/worksheet.php, accessed on 15 August 2024).

### 2.3. Depressive Symptoms Assessment

Depressive symptoms were assessed using the Japanese version of CES-D [21]. The total CES-D score ranged from 0 to 60, with higher scores indicating more symptoms, weighted by the frequency of occurrence during the past week [22]. This score was used as a continuous variable in the analyses.

### 2.4. Dietary Assessment

Dietary habits during the preceding month were assessed using the Diet History Questionnaire (DHQ) developed by Sasaki et. al. [23,24,25,26,27]. DHQ is a structured questionnaire consisting of more than 400 questions from which dietary intake derived from 149 foods and over 30 nutrients are calculated. DHQ has been tested for validation by several methods (using biomarkers or other diet assessors) in Japanese adults and pregnant women [23,24,25,26,27]. Estimates of daily food intake, energy, and selected nutrients were calculated using an ad hoc computer algorithm for the DHQ based on the Standard Tables of Food Composition in Japan. A database for the population comprised of individual data is then produced and used for statistical analyses. Dietary intake values were energy-adjusted using the density method (i.e., amount per 100 kcal of energy for foods).

### 2.5. Fetal Development Assessment

Standard deviation scores (SDSs) for BW, BH, and birth head circumference were calculated using nordiFIT^®^ (Novo Nordisk Pharma, Tokyo, Japan). This software calculates SDSs for BW, BH, and birth head circumference by gestational age, according to sex-specific standards based on the Japanese population [28].

### 2.6. Statistical Analyses

Linear regression models were used to assess the relationship between CES-D score, blood cortisol levels, and blood MDA levels with maternal food type/nutrient intake. Multivariate linear regression models were used to adjust for a priori selected potential confounders: maternal age and BMI before pregnancy. To avoid overfitting, given the small sample size, we restricted the number of total covariates in the model so that for each covariate there will be at least 20 events [29]. A *p*-value of <0.05 was considered statistically significant. Measurement data largely below and largely above the detection limits of the measurement kits, as well as outliers detected using studentized residuals, were excluded from the regression analyses. All tests were performed using JMP^®^, Version 18.0.1 (SAS Institute Inc., Cary, NC, USA).

## 3. Results

Characteristics of the study subjects are shown in Table 1. Among the total number of participating mothers, 5.2% had hypertension, 3.4% had gestational diabetes, 12.1% consumed alcohol during pregnancy, and all delivered via Cesarean section. Among the total number of infants born, 12.1% were low birth weight (<2500 g).

The estimated daily intake of different food types and nutrients is shown in Table 2. The relationship between intake of different food types and nutrients during pregnancy and oxidative stress markers is shown in Table 3. Maternal serum MDA levels were inversely associated with the consumption of nuts (β = −0.40, 95%CI: −0.72~−0.09, *p* = 0.01, R^2^ adjusted: 0.15) and positively associated with the consumption of vitamin B2 (β = 2.43, 95%CI: 0.09~4.77, *p* = 0.04, R^2^ adjusted: 0.11) and manganese (β = 0.44, 95%CI: 0.06~0.83, *p* = 0.02, R^2^ adjusted: 0.13). Dietary intake during pregnancy was not associated with cord plasma MDA, PON-1, or PAF-AH levels. No significant association was observed between maternal serum MDA and cord plasma MDA levels (bivariate analysis: β = 0.02, 95%CI: −0.38~0.43, *p* = 0.90, R^2^: 0.00).

There were no significant associations between cord plasma MDA level and infant BWSDS (bivariate analysis: β = −0.03, 95%CI: −0.66~0.59, *p* = 0.91, R^2^: 0.00), BHSDS (bivariate analysis: β = −0.23, 95%CI: −0.76~0.30, *p* = 0.39, R^2^: 0.01), or birth head circumference SDS (bivariate analysis: β = 0.36, 95%CI: −0.21~0.93, *p* = 0.21, R^2^: 0.03).

The relationship between intake of different food types and nutrients during pregnancy and CES-D score or blood cortisol level is shown in Table 4. Fruit consumption was positively associated with CES-D score (β = 0.35, 95%CI: 0.03~0.66, *p* = 0.03, R^2^ adjusted: 0.07). Intakes of beans (β = −3.37, 95%CI: −6.57~−0.17, *p* = 0.04, R^2^ adjusted: 0.03), vitamin B1 (β = −738.92, 95%CI: −1441.35~−36.49, *p* = 0.04, R^2^ adjusted: 0.03), vitamin B6 (β = −562.21, 95%CI: −1110.72~−13.71, *p* = 0.04, R^2^ adjusted: 0.02), vitamin C (β = −4.75, 95%CI: −8.23~−1.27, *p* = 0.009, R^2^ adjusted: 0.08), iron (β = −106.63, 95%CI: −201.27~−11.99, *p* = 0.03, R^2^ adjusted: 0.04), and copper (β = −863.31, 95%CI: −1519.57~−207.04, *p* = 0.01, R^2^ adjusted: 0.07) were inversely associated with maternal serum cortisol levels, while the association was positive for intake of dairy products (β = 1.45, 95%CI: 0.50~2.39, *p* = 0.003, R^2^ adjusted: 0.10). There was no significant association between maternal serum cortisol levels and CES-D score (bivariate analysis: β = −0.01, 95%CI: −0.03~0.01, *p* = 0.31, R^2^: 0.02), nor between maternal serum MDA levels and CES-D score (bivariate analysis: β = −0.64, 95%CI: −7.29~6.00, *p* = 0.85, R^2^: 0.00). Cord plasma cortisol levels were inversely associated with the consumption of other vegetables (β = −2.89, 95%CI: −5.21~−0.57, *p* = 0.02, R^2^ adjusted: 0.09).

## 4. Discussion

In this study, we have shown that diet during late pregnancy is associated with maternal oxidative stress status, stress, and depression status. Despite its association with cord blood cortisol levels, maternal diet during late pregnancy was not linked to fetal oxidative stress status, which, in turn, was not associated with fetal physical development.

Our results are consistent with previous reports on the association between dietary intake during pregnancy and maternal oxidative stress levels. In the Korean Mothers and Children’s Environmental Health (MOCEH) study, a maternal dietary pattern with high intakes of grains, green/yellow and light-colored vegetables, kimchi, legumes, fruits, meat, eggs, fish, seaweeds, tofu/soymilk, yogurt, and nuts was inversely related to high-sensitivity C-reactive protein in the blood and MDA concentrations in the urine [11]. This aligns with our finding that maternal serum MDA levels were inversely associated with the consumption of nuts. This could be because nuts are rich in antioxidants, which are involved in the reduction of free radical production, thereby reducing oxidative stress [30,31]. Additionally, certain phytochemicals in nuts can up-regulate the Nrf2 pathway, which stimulates the antioxidant response element gene transcription and the various antioxidant enzymes it encodes including GPx that suppresses oxidative stress [32]. We found positive associations between maternal serum MDA levels and the consumption of vitamin B2 and manganese, suggesting that the appropriate intake of nutrients should be carefully considered. In contrast to previous reports, where an increase in the consumption of vitamin C was associated with an increase in maternal serum MDA levels [10] or inversely correlated with urine MDA levels [12], our result showed no association between vitamin C intake in late pregnancy and maternal serum MDA levels. This could be due to the difference in vitamin C intake between the studied populations, where the consumption amount in our study (mean ± standard deviation [SD]: 4.9 ± 1.9 mg/100 kcal) was roughly half of that in the previous study (mean ± SD: 9.9 ± 4.7 mg/100 kcal) [10]. Although a previous report showed that cord blood MDA levels were associated with maternal blood MDA levels and dietary intake of vitamin A [10], our results showed no such relationships. It is of note that the consumption amount of vitamin A in the population of a previous study (mean ± SD: 49.0 ± 16.2 µg/100 kcal) [10] was approximately twice the amount in our population (mean ± SD: 25.7 ± 12.4 µg/100 kcal).

A previous report indicated that infants born via vaginal delivery or emergency Cesarean section had higher cord blood MDA concentrations compared with those born via elective Cesarean section [33]. An inverse correlation was detected between head circumference and MDA levels in the cord blood of vaginal-delivery-born infants, while no statistically significant correlation was identified between head circumference and MDA levels in the cord blood of elective Cesarean or emergency Cesarean section infants [33]. Another report showed that infants born smaller than their gestational age had higher cord blood MDA concentrations and lower superoxide dismutase (SOD) concentrations compared with infants born larger than their gestational age [34]. In this study, no correlation was found between cord blood MDA concentrations and birth weight SDS, birth height SDS, or birth head circumference SDS. This may be because this study included many full-term infants born by scheduled Cesarean section rather than sick infants. The difference in cord blood MDA concentrations between scheduled Cesarean and vaginal delivery could not be evaluated, as all infants in this study were born via scheduled Cesarean section.

We found that increased fruit intake during late pregnancy was associated with a higher CES-D score. However, it is important to note that most participants who completed the questionnaire (87.7%) had scores below 16 (within the range of 0–60), which is considered “not high” on the scale [22]. Regarding blood cortisol levels, we found that intake of beans, vitamin B1, vitamin B6, vitamin C, iron, and copper during late pregnancy was inversely associated with maternal serum cortisol levels. This is supported by previous reports on the effects of trace elements, such as lithium, vitamin B6, vitamin B12, and folic acid, on the facilitation of gamma-aminobutyric acid (GABA) system activity, which, in turn, reduces the secretion of corticoliberin and, thus, reduces cortisol levels [35]. Furthermore, GABA is naturally found in beans [36]. In contrast to previous reports suggesting that the consumption of fermented milk products reduces cortisol levels [35], our result showed a positive association between dairy consumption during late pregnancy and maternal serum cortisol levels. We found that the level of cortisol in cord plasma was inversely associated with maternal intake of other vegetables during late pregnancy. This is consistent with previous studies showing that offspring of mothers who had high meat/fish and low green vegetable consumption in late pregnancy had increased fasting plasma cortisol concentrations [37] and greater cortisol secretion in response to psychological stress in adulthood [38].

Compared to previous studies, where dietary assessments spanned the entire pregnancy, we focused on collecting data during late pregnancy, allowing for a more targeted interpretation of the importance of nutritional intake during this period. Our data were also adjusted for potential confounders using multivariate linear regression. However, certain limitations should be noted, including the inability to examine differences in cord blood MDA concentrations between delivery methods, such as vaginal delivery and Cesarean section, as well as the fact that this study did not include sick infants, such as premature or low birth weight infants. In this study, we handled the dietary intake of certain food types or nutrients as a continuous variable in the analyses, but we did not address subgroups, which could offer some additional evidence. The dietary survey was based on the participants’ recall of their diet during the previous month, which could be subject to bias. The study population mostly included women at around 37–40 weeks of gestation, which might limit the generalizability of this study. With a small sample size, the findings from this study should be considered preliminary and require replication, including research in other study populations and an evaluation of the underlying mechanisms of action. Despite this study’s limitations, our findings encourage further research by those who aim toward the refinement of dietary guidelines for pregnant women and towards giving direction for recommendations for expecting mothers.

## 5. Conclusions

In the present study, we showed that diet during late pregnancy influences maternal health through its association with blood MDA and cortisol levels and impacts infant development through its association with cord blood cortisol levels. Of note are the inverse associations (1) between nut consumption and maternal blood MDA levels, (2) between intakes of beans, vitamin B1, vitamin B6, vitamin C, iron, and copper and maternal blood cortisol levels, and (3) between the consumption of other vegetables and cord blood cortisol levels. We acknowledge the necessity of further studies in other populations, as well as research into the underlying mechanisms of action, to deepen our understanding of these relationships. Especially important are longitudinal studies, from which long-term outcomes in the born children could be evaluated. Our findings encourage further research that aims towards the refinement of dietary guidelines for pregnant women and recommendations for expecting mothers.

## Figures and Tables

**Table 1:** Baseline characteristics of study participants.

Participants	Characteristics	Total (n = 58) ^a^
Mothers	Age (years)	35 (31–38)
	Delivery method (%)	
	Vaginal	0
	Cesarean section	100
	BMI before pregnancy (kg/m^2^)	22.4 (20.3–24.0)
	Placenta weight (g)	567.0 (476.3–628.5)
	Hypertension (%)	5.2
	Gestational diabetes (%)	3.4
	Cigarette smoking during pregnancy (%)	0
	Alcohol intake during pregnancy (%)	12.1
Infants	Number of weeks of gestation (weeks)	37 (37–38)
	Birth weight (g)	2848.0 (2625.5–3065.5)
	Birth weight SDS ^b^	0.18 (−0.34–0.64)
	Birth height (cm)	49.0 (48.0–50.0)
	Birth height SDS ^b^	0.44 (0.19–0.91)
	Birth head circumference (cm)	33.3 (32.5–34.0)
	Birth head circumference SDS ^b^	0.31 (−0.39–0.82)
	Premature infants (%)	0
	Low birth weight infants (<2500 g) (%)	12.1
	Neonatal asphyxia (%)	0

^a^ Values are medians (interquartile range) for continuous variables and percentage of subjects for categorical variables. ^b^ SDS: standard deviation score.

**Table 2:** Estimated daily food or nutrient intake during pregnancy.

Variable	Total (n = 58) ^a^ Mean ± SD ^b^
Energy (kcal)	1843 ± 595
Nuts (g/100 kcal)	0.06 ± 0.12
Potatoes (g/100 kcal)	1.36 ± 0.83
Beans (g/100 kcal)	2.60 ± 2.14
Fruit (g/100 kcal)	5.62 ± 4.53
Green and yellow vegetables (g/100 kcal)	4.62 ± 3.05
Other vegetables (g/100 kcal)	5.21 ± 3.46
Mushrooms (g/100 kcal)	0.59 ± 0.48
Seaweeds (g/100 kcal)	0.47 ± 0.66
Seafoods (g/100 kcal)	1.88 ± 0.97
Meats (g/100 kcal)	4.23 ± 1.94
Eggs (g/100 kcal)	1.27 ± 1.09
Dairy (g/100 kcal)	9.53 ± 6.99
Vitamin A (μg/100 kcal)	25.74 ± 12.44
Vitamin D (μg/100 kcal)	0.25 ± 0.11
Vitamin E (mg/100 kcal)	0.42 ± 0.10
Vitamin K (μg/100 kcal)	13.07 ± 6.85
Vitamin B1 (mg/100 kcal)	0.05 ± 0.01
Vitamin B2 (mg/100 kcal)	0.07 ± 0.02
Niacin (mg/100 kcal)	0.70 ± 0.18
Vitamin B6 (mg/100 kcal)	0.05 ± 0.01
Vitamin B12 (μg/100 kcal)	0.22 ± 0.10
Folate (μg/100 kcal)	14.75 ± 4.24
Pantothenic acid (mg/100 kcal)	0.31 ± 0.05
Vitamin C (mg/100 kcal)	4.92 ± 1.93
Na (mg/100 kcal)	214.58 ± 53.82
K (mg/100 kcal)	111.42 ± 21.37
Ca (mg/100 kcal)	29.32 ± 8.26
Mg (mg/100 kcal)	11.77 ± 2.23
P (mg/100 kcal)	50.49 ± 8.10
Fe (mg/100 kcal)	0.35 ± 0.07
Zn (mg/100 kcal)	0.39 ± 0.05
Cu (mg/100 kcal)	0.05 ± 0.01
Mn (mg/100 kcal)	0.21 ± 0.10

^a^ Food intake level was adjusted for total energy intake using the density method. ^b^ SD: standard deviation.

**Table 3:** Relation between food or nutrient intake during pregnancy and oxidative stress markers levels ^a^.

Variable ^b^	Maternal Serum MDA ^c^ (n = 51) β, *p*		Cord Plasma MDA ^c^ (n = 54) β, *p*	Cord Plasma PON-1 ^d^ (n = 58) β, *p*	Cord Plasma PAF-AH ^e^ (n = 49) β, *p*
	Crude	Adjusted ^f^	Crude	Crude	Crude
Nuts (g/100 kcal)	−0.33, 0.03 *	−0.40, 0.01 *	0.49, 0.24	−13.72, 0.93	11.15, 0.68
Potatoes (g/100 kcal)	0.01, 0.72	−	0.04, 0.50	−3.71, 0.88	−0.12, 0.98
Beans (g/100 kcal)	0.00, 0.65	−	0.00, 0.89	−12.44, 0.18	−0.62, 0.74
Fruit (g/100 kcal)	0.00, 0.97	−	0.01, 0.48	0.71, 0.87	0.09, 0.91
Green and yellow vegetables (g/100 kcal)	0.01, 0.41	−	0.02, 0.21	−0.59, 0.93	−1.52, 0.17
Other vegetables (g/100 kcal)	−0.01, 0.23	−	−0.01, 0.49	−3.95, 0.49	−1.30, 0.27
Mushrooms (g/100 kcal)	0.04, 0.38	−	−0.08, 0.46	−62.60, 0.13	−0.72, 0.92
Seaweeds (g/100 kcal)	0.01, 0.62	−	−0.11, 0.17	−33.41, 0.26	−10.61, 0.16
Seafoods (g/100 kcal)	−0.01, 0.77	−	0.03, 0.64	3.53, 0.86	−1.65, 0.65
Meats (g/100 kcal)	−0.02, 0.03 *	−0.02, 0.07	0.02, 0.36	9.45, 0.35	−1.39, 0.48
Eggs (g/100 kcal)	0.03, 016	−	−0.02, 0.76	0.27, 0.99	−0.96, 0.76
Dairy (g/100 kcal)	0.00, 0.29	−	0.00, 0.64	−0.97, 0.73	0.10, 0.85
Vitamin A (μg/100 kcal)	0.00, 0.94	−	0.00, 0.39	1.16, 0.47	0.40, 0.14
Vitamin D (μg/100 kcal)	0.02, 0.92	−	0.40, 0.44	155.19, 0.41	−23.32, 0.47
Vitamin E (mg/100 kcal)	−0.35, 0.09	−	−0.11, 084	−117.12, 0.55	−18.44, 0.66
Vitamin K (μg/100 kcal)	0.00, 0.94	−	0.00, 0.77	0.36, 0.90	−0.66, 0.30
Vitamin B1 (mg/100 kcal)	−2.53, 0.20	−	9.52, 0.06	1378.69, 0.47	−487.52, 0.16
Vitamin B2 (mg/100 kcal)	2.42, 0.049 *	2.43, 0.04 *	−2.05, 0.53	366.97, 0.77	−86.38, 0.70
Niacin (mg/100 kcal)	−0.14, 0.20	−	0.33, 0.26	57.31, 0.60	−23.27, 0.30
Vitamin B6 (mg/100 kcal)	−0.38, 0.80	−	1.54, 0.70	−627.64, 0.68	−292.35, 0.31
Vitamin B12 (μg/100 kcal)	0.03, 0.87	−	0.19, 0.73	75.89, 0.71	17.11, 0.64
Folate (μg/100 kcal)	0.01, 0.05	−	0.00, 0.92	−1.72, 0.71	−1.06, 0.21
Pantothenic acid (mg/100 kcal)	0.41, 0.31	−	−0.17, 0.87	151.40, 0.70	−30.15, 0.70
Vitamin C (mg/100 kcal)	0.02, 0.07	−	0.02, 0.55	−4.72, 0.65	−2.91, 0.11
Na (mg/100 kcal)	0.00, 0.63	−	0.00, 0.88	−0.10, 0.78	−0.03, 0.71
K (mg/100 kcal)	0.00, 0.69	−	0.00, 0.95	−0.08, 0.93	−0.17, 0.34
Ca (mg/100 kcal)	0.00, 0.27	−	0.00, 0.57	−1.11, 0.64	0.26, 0.57
Mg (mg/100 kcal)	0.00, 0.68	−	0.00, 0.96	−4.85, 0.59	−1.48, 0.38
P (mg/100 kcal)	0.00, 0.67	−	0.00, 0.73	−0.03, 0.99	0.10, 0.84
Fe (mg/100 kcal)	0.33, 0.23	−	0.05, 0.94	−41.68, 0.88	−47.40, 0.34
Zn (mg/100 kcal)	0.16, 0.70	−	0.63, 0.55	208.95, 0.60	−33.46, 0.66
Cu (mg/100 kcal)	1.77, 0.36	−	1.66, 0.74	346.55, 0.86	−351.15, 0.28
Mn (mg/100 kcal)	0.42, 0.04 *	0.44, 0.02 *	−0.18, 0.73	−42.58, 0.83	−38.57, 0.27

^a^ Data largely out of detection limit and outliers (detected using studentized residuals) were excluded from the regression analyses. ^b^ Food intake level was adjusted for total energy intake using the density method. ^c^ MDA: malondialdehyde. ^d^ PON-1: paraoxonase-1. ^e^ PAF-AH: platelet-activating factor-acetylhydrolase. ^f^ Adjusted for age and BMI before pregnancy. * *p* < 0.05.

**Table 4:** Relationship between food or nutrient intake during pregnancy and CES-D score and blood cortisol level ^a^.

Variable ^b^	CES-D Score (n = 55) β, *p*		Maternal Serum Cortisol (n = 56) β, *p*		Cord Plasma Cortisol (n = 56) β, *p*	
	Crude	Adjusted ^c^	Crude	Adjusted ^c^	Crude	Adjusted ^d^
Nuts (g/100 kcal)	0.97, 0.87	−	11.32, 0.68	−	−13.26, 0.66	−
Potatoes (g/100 kcal)	−0.96, 0.28	−	−6.42, 0.12	−	1.82, 0.69	−
Beans (g/100 kcal)	0.14, 0.68	−	−3.30, 0.04 *	−3.37, 0.04 *	−3.19, 0.07	−
Fruit (g/100 kcal)	0.37, 0.02 *	0.35, 0.03 *	−0.19, 0.80	−	0.37, 0.67	−
Green and yellow vegetables (g/100 kcal)	−0.23, 0.32	−	−2.14, 0.06	−	−1.11, 0.38	−
Other vegetables (g/100 kcal)	0.00, 1.00	−	−1.87, 0.08	−	−2.42, 0.04 *	−2.89, 0.02 *
Mushrooms (g/100 kcal)	−0.28, 0.86	−	4.70, 0.52	−	−4.06, 0.61	−
Seaweeds (g/100 kcal)	−0.51, 0.64	−	−3.67, 0.48	−	−9.37, 0.10	−
Seafoods (g/100 kcal)	0.33, 0.67	−	−4.59, 0.20	−	−3.56, 0.37	−
Meats (g/100 kcal)	−0.47, 0.21	−	−1.51, 0.45	−	−0.58, 0.79	−
Eggs (g/100 kcal)	−0.14, 0.83	−	−1.65, 0.63	−	5.33, 0.15	−
Dairy (g/100 kcal)	0.07, 0.50	−	1.42, 0.003 **	1.45, 0.003 **	1.01, 0.06	−
Vitamin A (μg/100 kcal)	−0.03, 0.58	−	0.02. 0.96	−	−0.21, 0.49	−
Vitamin D (μg/100 kcal)	4.42, 0.53	−	−37.74, 0.24	−	−13.69, 0.70	−
Vitamin E (mg/100 kcal)	0.64, 0.94	−	−24.53, 0.52	−	−35.12, 0.41	−
Vitamin K (μg/100 kcal)	−0.17, 0.10	−	−0.90, 0.11	−	−1.18, 0.05	−
Vitamin B1 (mg/100 kcal)	−80.82, 0.29	−	−696.29, 0.04 *	−738.92, 0.04 *	−188.13, 0.62	−
Vitamin B2 (mg/100 kcal)	6.48, 0.89	−	132.79, 0.54	−	232.19, 0.33	−
Niacin (mg/100 kcal)	−6.80, 0.10	−	−37.7, 0.08	−	−31.44, 0.18	−
Vitamin B6 (mg/100 kcal)	−37.61, 0.52	−	−539.44, 0.04 *	−562.21, 0.04 *	−381.81, 0.20	−
Vitamin B12 (μg/100 kcal)	1.53, 0.84	−	−10.76, 0.76	−	−2.40, 0.95	−
Folate (μg/100 kcal)	−0.08, 0.63	−	−1.41, 0.08	−	−1.16, 0.19	−
Pantothenic acid (mg/100 kcal)	−12.89, 0.43	−	28.05, 0.68	−	26.26, 0.73	−
Vitamin C (mg/100 kcal)	0.40, 0.30	−	−4.66, 0.01 *	−4.75, 0.009 **	−1.69, 0.40	−
Na (mg/100 kcal)	0.00, 0.92	−	−0.07, 0.27	−	−0.13, 0.06	−
K (mg/100 kcal)	−0.01, 0.72	−	−0.16, 0.35	−	−0.09, 0.65	−
Ca (mg/100 kcal)	0.05, 0.59	−	0.73, 0.08	−	0.29, 0.53	−
Mg (mg/100 kcal)	−0.34, 0.32	−	−2.09, 0.19	−	−2.29, 0.19	−
P (mg/100 kcal)	−0.05, 0.62	−	0.12, 0.77	−	−0.06, 0.89	−
Fe (mg/100 kcal)	−0.69, 0.95	−	−105.53, 0.03 *	−106.63, 0.03 *	−87.32, 0.10	−
Zn (mg/100 kcal)	−13.16, 0.41	−	−110.12, 0.12	−	−27.48, 0.73	−
Cu (mg/100 kcal)	−33.49, 0.65	−	−833.31, 0.01 *	−863.31, 0.01 *	−667.48, 0.07	−
Mn (mg/100 kcal)	−4.81, 0.51	−	−25.29, 0.48	−	−2.32, 0.95	−

^a^ Outliers (detected using studentized residuals) were excluded from the regression analyses. ^b^ Food intake level was adjusted for total energy intake using the density method. ^c^ Adjusted for age and BMI before pregnancy. ^d^ Adjusted for maternal age and maternal BMI before pregnancy. * *p* < 0.05, ** *p* < 0.01.

## Data Availability

The datasets analyzed during the current study are not publicly available because the steering committee and the participants did not approve unrestricted data sharing.
